# Chemerin Effect on the Endometrial Proteome of the Domestic Pig during Implantation Obtained by LC-MS/MS Analysis

**DOI:** 10.3390/cells11071161

**Published:** 2022-03-30

**Authors:** Kinga Orzechowska, Kamil Dobrzyń, Marta Kieżun, Agata Malinowska, Bianka Świderska, Tadeusz Kamiński, Nina Smolińska

**Affiliations:** 1 Department of Animal Anatomy and Physiology, Faculty of Biology and Biotechnology, University of Warmia and Mazury in Olsztyn, Oczapowskiego St. 1A, 10-719 Olsztyn, Poland; kinga.bors@uwm.edu.pl (K.O.); marta.kiezun@uwm.edu.pl (M.K.); tkam@uwm.edu.pl (T.K.); 2 Department of Zoology, Faculty of Biology and Biotechnology, University of Warmia and Mazury in Olsztyn, Oczapowskiego St. 5, 10-718 Olsztyn, Poland; kamil.dobrzyn@uwm.edu.pl; 3 Mass Spectrometry Laboratory, Institute of Biochemistry and Biophysics PAS, 5a Pawińskiego St., 02-106 Warsaw, Poland; esme@ibb.waw.pl (A.M.); bianka.swiderska@gmail.com (B.Ś.)

**Keywords:** chemerin, pig, implantation, uterus, proteome, early pregnancy, DRPs

## Abstract

Chemerin (CHEM) is a hormone mainly expressed in adipocytes involved in the regulation of energy homeostasis and inflammatory response. CHEM expression has been demonstrated in the structures of the porcine hypothalamic-pituitary-gonadal axis, as well as in the uterus, trophoblasts and conceptuses of pigs. In this study, we performed high-throughput proteomic analyses (liquid chromatography with tandem mass spectrometry, LC-MS/MS) to examine the influence of CHEM (400 ng/mL) on differentially regulated proteins (DRPs) in the porcine endometrial tissue explants during implantation (15 to 16 days of gestation). Among all 352 DRPs, 164 were up-regulated and 188 were down-regulated in CHEM-treated group. DRPs were assigned to 47 gene ontology (GO) terms (*p*-adjusted < 0.05). Validation of four DRPs (IFIT5, TGFβ1, ACO1 and PGRMC1) by Western blot analysis confirmed the veracity and accuracy of the LC-MS/MS method used in the present study. We suggest that CHEM, by modulating various protein expressions, takes part in the endometrial cell proliferation, migration and invasion at the time of implantation. It also regulates the endometrial immune response, sensitivity to P4 and the formation of new blood vessels. Additionally, CHEM appears to be an important factor involved in endothelial cell dysfunction during the pathogenesis of preeclampsia. The identification of a large number of DRPs under the influence of CHEM provides a valuable resource for understanding the molecular mechanisms of this hormone action during implantation, which is a prerequisite for better control of pig reproduction.

## 1. Introduction

Secretory products from maternal uterine epithelia and precise cell-specific molecular signaling between embryos and the endometrium are critical to conceptus growth and development, pregnancy recognition signaling and implantation [1]. Regulation of the uterine receptivity and conceptus adhesion to the endometrium is critical for placentation. Alternations of these signaling cascades can lead to an abnormal exchange of nutrients and pregnancy failure. A comprehensive understanding of the molecular mechanisms governing conceptus implantation is critical for the improvement of the fertility and reproductive health of pigs [2,3]. The roles of various feto-maternal signals, such as progesterone, estrogens, prostaglandins, interleukins, interferons and growth factors have been demonstrated to be involved in the implantation process [4]. In addition to the above molecules, there are many more unique proteins within the uterine environment of pigs that take part in the establishment of pregnancy. For example, studies indicated that thyroid hormones, thyroid-stimulating hormone receptor and thyroid hormone receptors (α1, α2, and β1) are present in the endometrium and influence implantation taking part in crosstalk between the competent blastocyst and the receptive endometrium [5,6]. Plenty of studies have shown that the hormones produced by adipose tissue, like leptin [7] and chemerin [8], also take part in the control of reproduction.

Chemerin (CHEM) is a hormone mainly expressed in adipocytes [9]. CHEM exerts its physiological functions through binding to three G protein-coupled receptors: chemokine like receptor 1 (CMKLR1), G protein-coupled receptor 1 (GPR1) and CC motif chemokine receptor-like 2 (CCRL2) [10]. It plays a pleiotropic role as a cytokine and adipokine (a hormone produced by the adipose tissue). CHEM regulates inflammatory response and energy homeostasis [11]. Recent studies indicated that CHEM also takes part in the control of reproduction by the modulation of, among others, uterine and ovarian steroidogenesis during both the early pregnancy and the estrous cycle [8,12,13,14]. CHEM expression has been demonstrated in the structures of the porcine hypothalamic-pituitary-gonadal axis, as well as in the uterus, trophoblasts and conceptuses of pigs [15,16,17,18,19].

CHEM, as a cytokine and adipokine, is a very interesting research object and can play an essential role in the implantation process. Gudelska et al. [19] confirmed that the implantation/embryo attachment was the stage of the highest CHEM protein expression in the porcine endometrium, focusing our attention on the role of CHEM in this process. Moreover, Yang et al. [20] reported that the expression of CHEM in the human decidua correlates with early spontaneous abortion. The above reports suggest that CHEM may be an important factor in the maintenance of early pregnancy. However, there is a lack of information on the exact roles that CHEM may play during implantation.

Recent studies have shown molecular changes in the porcine endometrium during the peri-implantation period and revealed sixteen differentially expressed proteins involved in the recognition and establishment of early gestation in pigs [21]. It has been hypothesized that CHEM may affect the proteome of the porcine uterus during implantation, and consequently influence the production of many factors essential for the proper course of gestation. Accordingly, in this work, using the tandem mass tag (TMT)-based quantitative proteomics method based on TMT-isobaric mass tag labeling and liquid chromatography with tandem mass spectrometry (LC–MS/MS) analysis, we present for the first time the proteome profile of the porcine endometrial explants affected by CHEM during the peri-implantation period (days 15 to 16 of gestation).

## 2. Materials and Methods

### 2.1. Collection of Samples

According to the Polish (Journal of Laws, 2015; item 266) and European (Directive 2010/63/Eu) regulations on the protection of animals used for scientific or educational purposes, the experiments did not require the consent of the competent ethics committee for animal experiments. The present study was conducted on five female crossbreed pigs (Large White × Polish Landrace, seven to eight months of age and 120 to 130 kg in weight), acquired from a private breeding farm. The animals were on days 15 to 16 of pregnancy (the beginning of implantation). Females were verified for estrus behavior in the presence of a boar daily. Insemination, by natural mating, was conducted on days one to two of the estrous cycle. The day after coition was indicated as the first day of pregnancy. Uteri were gathered immediately after slaughter, placed in ice-cold sterile phosphate-buffered saline (PBS), supplemented with 100 IU/mL penicillin, 100 μg/mL streptomycin and carried to the laboratory on ice.

### 2.2. Tissue Cultures

Uterine tissue cultures were performed as described by Smolinska et al. [22]. The explants were preincubated for 2 h at a rocking platform in a water bath at 37 °C (95% O_2_ and 5% CO_2_) and then treated for 24 h with human recombinant CHEM (cat. no. 268e10032; RayBiotech Life, Peachtree Corners, GA, USA) at the concentration of 400 ng/mL. Tissue explants incubated with medium alone were used as controls. The dose of CHEM was selected based on ULFs CHEM concentrations indicated in our previous study [19]. All cultures were performed in duplicates, in five independent experiments (*n* = 5). After incubation, the endometrial slices were frozen in liquid nitrogen and stored at −80 °C until the total protein extraction. The viability of tissue explants was monitored by quantifying lactate dehydrogenase (LDH) activity as we described in Dobrzyn et al. [23]. The average activity of LDH in the cultured tissue explants after the treatment period was 128.7 ± 35.6 U/L (0.67% of maximal release of LDH after the total endometrial cells destruction).

### 2.3. Protein Extraction

Protein extraction was performed by homogenizing 15–20 mg of endometrial tissue in 150–200 μL of lysis buffer (30 mM TrisHCl pH 8, 8 M urea and 2% CHAPS) on ice three times for 15 s using TissueRuptor (Qiagen, Hilden, Germany). Protein extracts were incubated on ice for 30 min and then centrifuged (10,000× *g* for 5 min at 4 °C). The supernatants were quantified using a Bradford dye-binding procedure with 0.5 mg/mL BSA as a standard. All analyses were performed in duplicate. Probes were stored at −80 °C for further analysis.

### 2.4. Protein’s Digestion

A proteomics analysis was performed by the Mass Spectrometry Laboratory at the Institute of Biochemistry and Biophysics of the Polish Academy of Sciences. 100 µg of each endometrial sample and pulled internal standard was diluted with 200 µL 8 M urea solution in a 200 mM triethylammonium bicarbonate buffer (TEAB, pH 8.5). Cysteine bridges were reduced by incubation with 20 mM tris(2-carboxyethyl)phosphine (TCEP) (1 h at 37 °C). Proteins were transferred into Vivacon 30 kDa molecular weight cut-off filter (Sartorius Stedim, Göttingen, Germany) and digested according to a filter-aided sample preparation (FASP) protocol with minor modifications. Samples were spun at 14,500× *g* for 30 min and washed with 100 µL urea solution before cysteine blocking by 15 min incubation with 50 mM s-methylmethanethiosulfonate (MMTS) at RT. Proteins were washed three times with 8 M urea buffer and 200 mM TEAB. After each addition, the samples were centrifuged until the cut-off filter was dry. Digestion was carried out using a trypsin/LysC mix (Promega, Madison, WI, USA) in 1:25 enzyme-to-protein ratio overnight at 37 °C. Peptides were eluted from spin filters by two washes with 200 mM TEAB and one wash with 500 mM NaCl. Dried peptides were resuspended in 85 µL of 200 mM TEAB buffer and labelled with TMT10plex (Thermo Fisher Scientific, Waltham, MA, USA) tags in 41 µL acetonitrile for 1 h on vortex. Internal standards were labelled with TMT10-126 label reagent. The reaction was quenched by the addition of 8 µL 5% hydroxylamine. The labelling efficiency was checked, and combined samples for each TMT set were desalted using two 30 mg Oasis HLB columns (Waters, Milford, MA, USA). Briefly, cartridges were preconditioned with 1 mL methanol and 1 mL MS-grade water. After the sample was loaded and rinsed with 1 mL water, peptides were eluted from columns with 400 µL of 80% acetonitrile (ACN) and 0.1% formic acid (FA). Aliquots were dried and resuspended in 500 µL 10 mM ammonium hydroxide.

### 2.5. Reversed-Phase Peptide Fractionation at High pH

TMT labelled peptides were fractionated using high-pH reverse-phase chromatography on an XBridge Peptide BEH C18 column (4.6 × 250 mm, 130 Å, 5 µm; Waters, Milford, MA, USA). Separation was performed at 1 mL/min flow rate for 27 min on a Waters Acquity UPLC H-class system. Mobile phases consisted of water (A), ACN (B) and 100 mM ammonium hydroxide solution (C). The percentage of phase C was kept at a constant 10% through the entire gradient. Fractions were collected every 1 min starting from the third minute of the run. The following gradient was applied: 3 to 5% solvent B for 0.5 min, 5 to 22% for 17 min, 22 to 28% for 2 min, 28 to 45% for 1.5 min, 45 to 90% for 0.5 min, 2.5 min isocratic hold at 90% and final column equilibration at 3% phase B for 3 min. The peptide elution profile was monitored at 214 nm by UV detector. Twenty-five fractions from each TMT set were dried in Speedvac and reconstituted in 100 uL 0.1% trifluoroacetic acid (TFA) and 2% acetonitrile before LC-MS/MS analysis.

### 2.6. Mass Spectrometry

Fractions were analyzed using an LC-MS/MS system comprised of an ACQUITY UPLC M-Class System (Waters, Milford, MA, USA) directly coupled to a QExactive mass spectrometer (Thermo Fisher Scientific, Waltham, MA, USA). Peptides were trapped on a C18 pre-column (180 µm × 20 mm; Waters, Milford, MA, USA) using 0.1% FA in water as a mobile phase and transferred to a nanoAcquity BEH C18 column (75 µm × 250 mm, 1.7 µm; Waters, Milford, MA, USA) using ACN gradient (0–35% ACN in 160 min) in the presence of 0.1% FA at a flow rate of 250 nL/min. Data acquisition was carried out using a data-dependent method with the top 12 precursors selected for MS2 analysis after collisional induced fragmentation (CID) with normalized collision energy (NCE) of 27. Full MS scans covering the mass range of 300–1600 m/z (mass-to-charge ratio) were acquired at a resolution of 70,000 with a maximum injection time of 60 ms and an AGC target value of 1e6. MS2 scans were acquired with a maximum injection time of 120 ms and an AGC target value of 5e5 with a resolution of 35,000. The isolation window was set to 1.2 m/z and a dynamic exclusion was set to 30 s.

### 2.7. Data Analysis

Offline recalibration, as well as peptides and proteins identification, was performed in the MaxQuant/Andromeda software suite (version 1.6.17.0) [24] using a *Sus scrofa* full Uniprot database (versions 2020_10). The search included tryptic and LysC-generated peptides, and Metylthio (C) was set as a fixed modification and oxidation (M) as a variable one. Reporter MS2 quantification was specified in order to obtain values for quantitative analysis. TMT 10plex correction factors were specified for each labelling set according to the manufacturer’s instructions. A reverse database was used for target/decoy statistical results validation. Protein groups along with quantitative data were further analyzed in Perseus (version 1.6.13) [25]. Hits from the reversed database and proteins only identified by site and contaminants were removed. TMT reporter values were normalized with internal reference scaling (IRS) [26]. Reporter intensities were log2 transformed and proteins with less than 3 values in at least one biological group were filtered out. Missing values were replaced with data from normal distribution (width 0.3, down shift 1.8) separately for each column. The significance threshold for *p*-value resulting from a Student’s t-test was 0.05 and the fold-change cut-off was ±1.2.

### 2.8. Network and Functional Analysis

To analyze the involvement of DRPs in the common biological processes, an enrichment ontology and pathway analysis was performed with the use of g:Profiler software (version e104_eg51_p15_3922dba) [27] based on GO [28], and KEGG [29] databases (*p*-adjusted < 0.05).

### 2.9. Validation of LC-MS/MS Results by Western Blot

In order to validate the LC-MS/MS results, we have performed a Western blot analysis for four chosen proteins (IFIT5, ACO1, TGFβ1, PGRMC1) selected from the list of proteins with the expression significantly changed under the influence of CHEM. The samples for Western blot analysis were homogenized on ice, with T-PER Tissue Protein Extraction Reagent (Thermo Fisher Scientific, Waltham, MA, USA) enriched with protease inhibitors (Sigma-Aldrich, Saint Louis, MO, USA). The lysates were cleared by centrifugation (10,000× *g*, 5 min, 4 °C). Western blot analysis was conducted according to Smolinska et al. [15], with modifications. Actin protein was used as a control for equal loading and to quantify the relative abundance of the examined proteins. 40 μg of total protein isolates were solubilized in a sample buffer (100 mM Tris-HCl, 4% SDS, 20% glycerol, 0.2% bromophenol blue and 200 mM dithiothreitol, pH = 6.8) and incubated for 3 min at 99.9 °C. Next, samples were separated by SDS-PAGE electrophoresis in a 12.5% polyacrylamide gel (100 V) and transferred onto a polyvinylidene fluoride membrane (PVDF) (Roche Diagnostics, Mannheim, Germany). A 5% BSA solution in TBST was used to block nonspecific binding. Next, the membranes were incubated at 4 °C overnight with specific primary antibodies: IFIT5 (1:500; ab220954; Abcam, Cambridge, UK), PGRMC1 (1:500; sc-393015; Santa Cruz Biotechnology, Dallas, TX, USA), ACO1 (1:1000; orb330127; Biorbyt, Cambridge, UK), TGFβ1 (1:500; PA1-29032; Thermo Fisher Scientific, Waltham, MA, USA), and actin (1:2000; A2066; Sigma-Aldrich, Saint Louis, MO, USA). After washing, membranes were incubated with HRP-conjugated secondary antibodies (1.5 h, RT): goat anti-rabbit IgG (AP156P; Merck Millipore, Burlington, MA, USA) for IFIT5, ACO1, TGFβ1 (1:5000) and actin (1:20,000), and goat anti-mouse IgG for PGRMC1 (1:5000; 115-035-003; Jackson ImmunoResearch, West Grove, PA, USA). The visualization of immunocomplexes was carried out using chemiluminescence HRP substrate (Merck Millipore, Burlington, MA, USA) according to the manufacturer’s protocol and visualized with an Azure 280 Imaging System (Azure Biosystems, Dublin, CA, USA). The results of the analysis were quantified by optical density (OD) analysis of immunocomplexes with Image Studio Lite v.5.2 (LI-COR, Lincoln, NE, USA). Data were presented as a ratio of examined protein relative to actin protein in arbitrary OD units. The normality of Western blot data distributions was confirmed using a Shapiro–Wilk test (*p* > 0.05), and the results were statistically checked by a Student’s *t*-test (*p* < 0.05) using Statistica software (Statsoft Inc., Tulsa, OK, USA). Data were presented as mean ± SEM (*n* = 5).

## 3. Results

### 3.1. Differentially Regulated Proteins and Functional Annotations (GO and KEGG)

**Figure 1:**
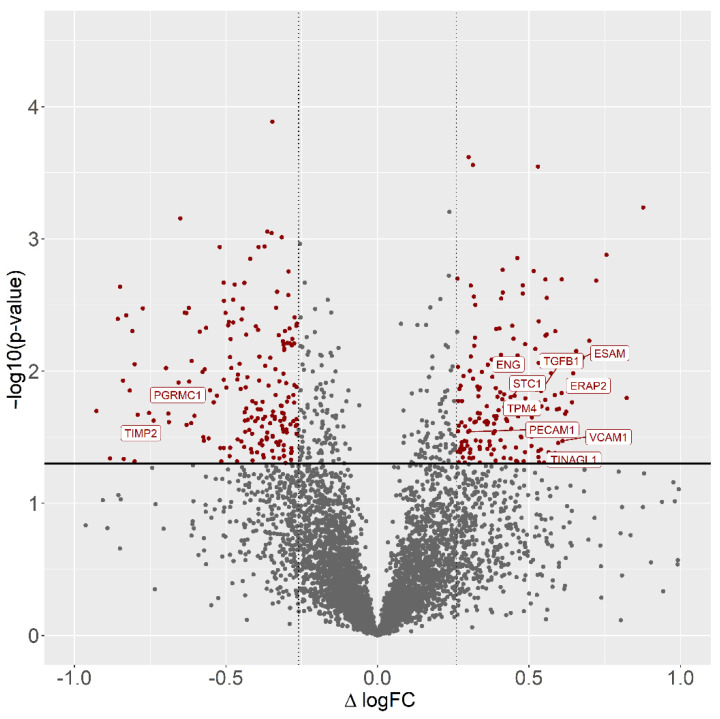
Volcano plot of the proteins identified by LC–MS/MS in CHEM vs. CTR comparison. The volcano plot shows the fold-change (*x*-axis) versus the significance (*y*-axis) of the identified differentially regulated proteins (DRPs) under the influence of CHEM. The significance (non-adjusted *p*-value) and the fold-change are converted to −Log10(*p*-value) and Log2(fold-change), respectively. The vertical and horizontal dotted lines show the cut-off of fold-change = ±1.2, and of *p*-value = 0.05, respectively. The chosen DRPs are labeled by the gene symbols.

A total of 1499 proteins were identified in the current study (Table S1). After relative quantification, filters (fold change cutoff 1.2 and *p* < 0.05) were applied to the results to obtain the final list of 352 differentially regulated proteins (DRPs). DRPs were visualized in the Volcano plot (Figure 1).

**Figure 2:**
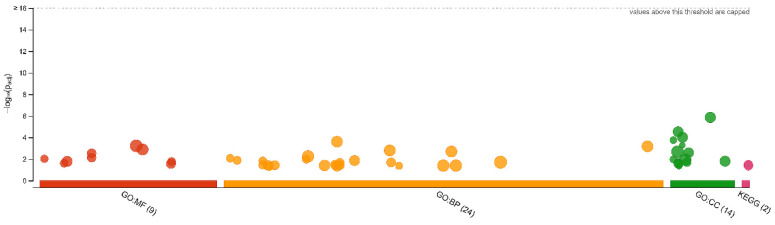
The g:Profiler analysis of differentially regulated proteins (DRPs). Manhattan plot that illustrates the enrichment analysis results. The functional terms are grouped and color-coded by data sources, i.e., molecular function (MF) are red, biological processes (BP) are orange, cellular components (CC) are green, and Kyoto Encyclopedia of Genes and Genomes (KEGG) are pink.

Among all DRPs, 164 were up-regulated and 188 were down-regulated in the CHEM-treated group (Table S2). The fold change values for DRPs ranged from −2.16 (SCD5) to 3.01 (HLA-DQB1). To discover possible functions of 352 annotated DRPs identified in the CHEM-treated porcine endometrial explants, the proteins were classified into three main categories: ‘biological processes’ (BP), ‘cellular components’ (CC) and ‘molecular function’ (MF) (Table S3). DRPs were assigned to 47 gene ontology (GO) terms (*p*-adjusted < 0.05); within these GO terms, 24 were ascribed to BP, 14 to CC and 9 to MF category (Figure 2).

**Figure 3:**
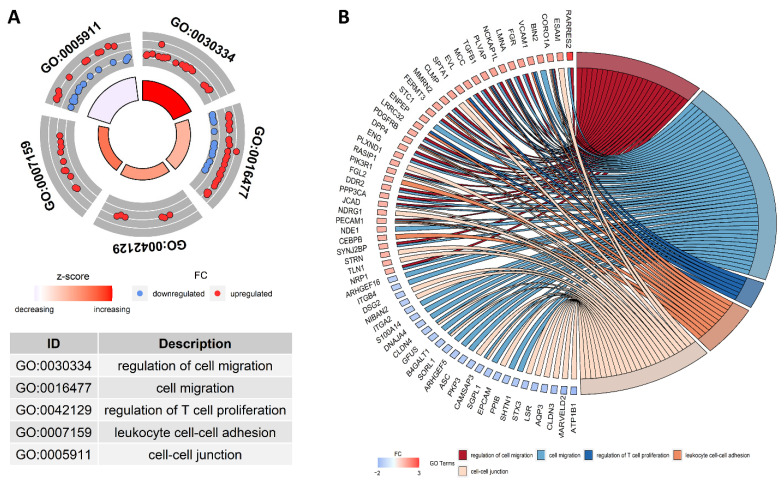
Visualization of the differentially regulated proteins (DRPs) determined to be statistically significant and their enrichment in the ontology terms detected by g:Profiler tool. (**A**) The circular plot illustrates the five most interesting GO biological processes enriched by DRPs. The outer track shows upregulated (red dots) and downregulated DRPs (blue dots) annotated in the particular GO terms. The inner track depicts the ratio of up- and downregulated proteins engaged in GO terms. A more intense red color shows the greater involvement of overexpressed proteins. (**B**) The circular plot shows the selected GO terms enriched by DRPs evaluated in the endometrium treated with CHEM.

Most of the DRPs from the BP category were allocated to ‘cell motility’ (GO:0048870; 43 DRPs), ‘localization of cell’ (GO:0051674; 43 DRGs), ‘cell migration’ (GO:0016477; 42 DRPs) and ‘carboxylic acid metabolic process’ (GO:0019752; 29 DRPs). Within the BP category, the most interesting DRPs were enriched to several subcategories: leukocyte cell-cell adhesion’ (GO:0007159; 11 DRPs), ‘regulation of cell migration’ (GO:0030334; 23 DRPs) and ‘regulation of T cell proliferation’ (GO:0042129; 6 DRPs) (Figure 3).

Most of the proteins assigned to the CC category were annotated to ‘cell junction’ (GO:0030054; 44 DRPs) and ‘anchoring junction’ (GO:0070161; 31 DRPs). The proteins were CC of e.g., cell-cell junction, cytoskeleton, cell leading edge, and polymeric cytoskeletal fiber.

The most enriched GO term in the MF category was ‘identical protein binding’ (GO:0042802; 59 DRPs). The MF aspect encompassed proteins annotated to ‘phospholipid binding’ (GO:0005543; 19 DRPs), ‘protein-containing complex binding’ (GO:0044877; 15 DRPs), ‘structural constituent of cytoskeleton’ (GO:0005200; 5 DRPs).

The Kyoto Encyclopedia of Genes and Genomes (KEGG) enrichment analysis revealed the modulation of two signaling pathways, activated by DRPs. The enrichment analysis qualified well-documented pathways that are linked with CHEM activation, e.g., ‘hypertrophic cardiomyopathy’ (KEGG:05410; 9 DRPs) and ‘dilated cardiomyopathy’ (KEGG:05414; 6 DRPs).

### 3.2. Western Blot

**Figure 4:**
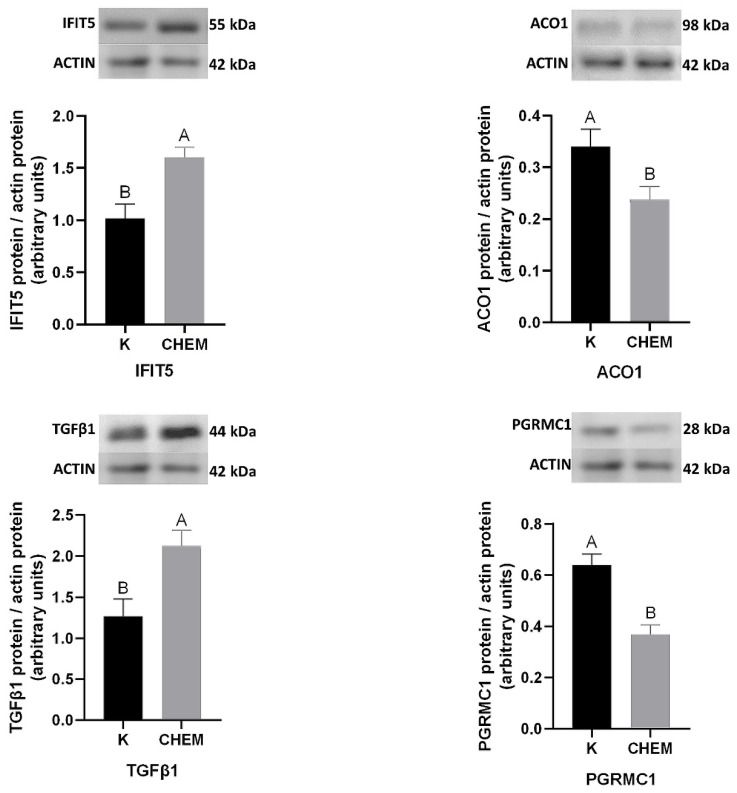
Western blot validation of the LC-MS results for differentially regulated proteins under the CHEM influence in the endometrium. Validation was performed for IFIT5, ACO1, TGFβ1 and PGRMC1 proteins with reference protein (actin) (*p*-value < 0.05). Data are presented as the mean ± standard error of the mean (n = 5). Bars with different letters are significantly different at *p* < 0.05. K—samples from the control groups; CHEM—samples from the CHEM-treated groups. IFIT5—Interferon Induced Protein With Tetratricopeptide Repeats 5; ACO1—Aconitase 1; TGFβ1—Transforming Growth Factor Beta 1; PGRMC1—membrane-associated progesterone receptor component 1.

To validate the obtained LC-MS/MS results, four DRPs were selected for western blot analysis. In the endometrial tissue treated in vitro with CHEM, when compared to the control, the abundances of Interferon Induced Protein With Tetratricopeptide Repeats 5 (IFIT5) and Transforming Growth Factor Beta 1 (TGFβ1) were up-regulated (*p* < 0.05). CHEM administration was related to a decreased protein content of Aconitase 1 (ACO1) and membrane-associated progesterone receptor component 1 (PGRMC1) (*p* < 0.05). The expression patterns of the selected DRPs agreed with the LC-MS/MS results (Figure 4). Validation of the results confirmed the veracity and accuracy of the LC-MS/MS method used in the present study.

## 4. Discussion

In the present study, for the first time, an LC-MS/MS analysis was performed to investigate DRPs in the cultured porcine endometrial explants exposed to CHEM (400 ng/mL) collected during the implantation period. In the current study, among all 352 DRPs, 164 were up-regulated and 188 were down-regulated in the CHEM-treated group. DRPs were assigned to 47 functional annotations. The above proteins were classified by GO functional annotation to processes connected to, among others, the regulation of cell migration and motility, the integrin-mediated signaling pathway, and the leukocyte cell-cell adhesion. Furthermore, we have confirmed the results obtained using high-throughput LC-MS/MS by the evolution of the CHEM effect on four chosen DRPs abundance in the porcine endometrium by the western blot method.

Progesterone (P_4_) is an important factor regulating the functioning of the endometrium. It participates in the preparation of the endometrium for implantation, and then maintains the pregnancy by inhibiting uterine muscle contractions, stimulating uterine growth and modulating the mother’s immune system [30]. P_4_ acts through the classical signaling pathway via nuclear P_4_ receptors (PRs) and also through non-classical signaling pathways [31]. In our in vitro study, one of the DRPs that down-regulated under the influence of CHEM was PGRMC1, which is a membrane-associated non-classical P_4_ receptor [32]. The PGRMC1 protein has been suggested to be important in early pregnancy because its expression is present in the human decidua at the maternal-fetal interface and associated membranes, with the highest expression being in the smooth muscle of placental blood vessels, villous capillaries, and in the syncytiotrophoblasts. In addition, it was shown that the expression of PGRMC1 protein depends on the stage of the estrous cycle and increases in endometrial cells undergoing steroid-dependent terminal differentiation [33]. Another study indicated that the mRNA level of *PGRMC1* significantly increased during gestation in the bovine endometrium [34]. It can be assumed that PGRMC1 is involved in cell differentiation. Lyzikova et al. [35] pointed to PGRMC1 as a potential factor involved in recurrent miscarriage. In the cases of recurrent miscarriage, endometrial protein expression of PGRMC1 is inversely correlated with infiltrated immune cells. It is suggested that PGRMC1 may regulate the immune response of the uterus [35]. The latest reports confirm that CHEM is involved in the regulation of steroidogenesis, including P_4_ secretion in the porcine endometrium [8]. In the light of these facts, we may assume that CHEM, except a direct impact on P_4_ secretion, in modulating endometrial PGRMC1 content may also be responsible for the proper sensitivity of tissues to the action of P_4_, which is necessary for the maintenance of pregnancy.

Regulation of the cell migration process is crucial in the control of trophoblast attachment [36]. Stanniocalcin 1 (STC1) is a glycoprotein assigned to the ‘regulation of cell migration’ category (GO:0030334), that reduces calcium levels and increases phosphate levels in the cells and tissues. STC1 is involved in many processes, including angiogenesis, mineral homeostasis, cell proliferation, inflammation, and apoptosis [37,38]. In our experiment, CHEM increased the level of this protein. Song et al. [37] showed that the STC1 gene and protein are present in the porcine endometrium and ULFs (uterine luminal flushing) during the estrus cycle and pregnancy. In addition, *STC1* mRNA expression has been shown to increase in the luminal epithelium (LE) between days 12 and 20 of pregnancy, possibly due to the increasing concentration of P_4_, which positively correlates with *STC1* expression [37]. Studies on the human endometrium demonstrated that *STC1* showed a similar expression with receptivity markers during the window of implantation [39]. The authors of the above studies suggest that STC1 may be a unique marker of implantation. CHEM, by stimulating STC1 content, may affect implantation through the effects on uterine receptivity.

The extracellular matrix (ECM) builds the intercellular network of collagen and proteoglycans through which it is possible to spread nutrients, metabolites, cytokines and growth factors. Degradation of ECM is essential for tissue development, morphogenesis and repair. Tissue inhibitors of metalloproteinases (TIMPs), by regulating the activity of matrix metalloproteinases (MMPs), are the most important mediators in ECM remodeling [40]. Our in vitro study has shown that CHEM diminished TIMP2 protein levels in the porcine endometrium during implantation. Ledgard et al. [41] indicated that TIMP2 protein was present in ULFs in lower amounts in pregnant compared with non-pregnant animals. The authors suggested that TIMP2 is involved in the maternal recognition of pregnancy [41]. On the other hand, another study has shown that the presence of conceptus induced endometrial *TIMP2* mRNA expression. Additionally, increased TIMP2 protein abundance in ULFs during embryo elongation was observed [42]. The presence of TIMP2 was also reported in the human decidua and placenta [43,44,45]. Tropomyosin alpha-4 chain (TPM4), up-regulated in this study by CHEM, is likewise associated with the cytoskeletal remodeling essential for pregnancy establishment. It has been shown that TPM4 protein is increased in the decidualized stromal cells during the late secretory phase of the menstrual cycle in women. The presence of this protein has also been confirmed in the decidual cells on the fetal-maternal interface in the first trimester of pregnancy [46]. Taking into consideration that CHEM modulated TIMP2 and TPM4 protein abundance, we can imply that CHEM may be involved in the control of endometrial remodeling during trophoblast invasion.

Subsequent DRPs identified in this study under CHEM influence, classified in the ‘regulation of cell migration’ category (GO:0030334), are TGFβ1 and its receptor Endoglin (ENG). TGFβ1 is one of the many growth factors identified in the porcine endometrium during the peri-implantation period [47,48]. Much research supports the finding that the porcine conceptus and endometrium are sources of secreted TGFβ1 during implantation, which suggests its possible roles in early embryo growth and maternal-conceptuses interactions [47,48,49,50,51]. The obtained results revealed the stimulatory effect of CHEM on TGFβ1 and ENG protein expression. Transgenic mice with inappropriate expression of TGFβ1 receptors in the uterus during early pregnancy were characterized by a delay in the attachment phase of implantation [52]. A study on the murine uterus has shown that the epithelial-derived TGFβ1 takes part in stromal differentiation at the time of implantation [53]. Other studies conducted on pregnant pigs on day 15 of gestation indicated that TGFβ1 plays also an immunosuppressive function [54]. Interestingly, Chadchan et al. [55] have observed up-regulated ENG protein and mRNA expression throughout the window of endometrial receptivity for mice embryo implantation. TGFβ1 and its receptor ENG are important for the normal implantation process, and we suggest that CHEM, by the stimulation of TGFβ1 and ENG levels, is an important regulating factor at the conceptus-maternal interface during the peri-implantation period in pigs.

In pigs, the maternal and fetal sides are connected by the placental microvasculature, thus there is a maternal-fetal exchange between the trophoblast and uterine luminal epithelium [56]. The dominant lymphocytes that accumulate at the interface between mother and fetus are the natural killers (NK). They play a key role in the maternal and fetal immune recognition and angiogenesis of the placenta. Any abnormalities in the migration of NK cells may lead to disturbances in the transformation of the uterine spiral arteries and adequate fetoplacental perfusion [57]. Carlino et al. [58] demonstrated that CHEM mRNA and protein are up-regulated during decidualization in the human endometrium and might contribute to NK cell accumulation and vascular remodeling during early gestation. Zhang et al. [59] proved that increased levels of TGFβ1 (described above) inhibit the activation of NK and their angiogenic properties. Moreover, epithelial cell adhesion molecule (EpCAM), down-regulated in this study by CHEM, is a transmembrane glycoprotein that promotes cell proliferation, migration and invasion [60,61]. Münz et al. [60] indicated that the inhibition of *EpCAM* expression strongly decreased proliferation and metabolism in human carcinoma cells. Studies on gliomas have shown that EpCAM overexpression significantly correlates with microvessel density, which indicates the participation of this protein in angiogenesis [62]. Other cancer studies also link EpCAM to the formation of new blood vessels [63,64,65]. Interestingly, EpCAM is expressed in the human [66] and mouse [67] endometrial epithelia and rat embryos [68]. Endothelial cell-selective adhesion molecule (ESAM) is the next cell adhesion molecule identified in this study to be up-regulated under the CHEM influence. ESAM is primarily expressed in the embryo-developing vasculature and takes part in cell-cell interactions [69]. It has been proven that tumor growth in ESAM knockout mice is retarded. The above phenomenon was related to less migratory and angiogenic activity [70]. Another enhanced cell adhesion molecule identified in this study is platelet endothelial cell adhesion molecule (PECAM1). PECAM1 is suggested to play a role in angiogenesis and leukocyte-endothelial interactions in the process of leukocyte margination during inflammation [71,72]. It is worth noting that EpCAM, ESAM, and PECAM1 are three of the 24 DRPs identified in this study, classified in the ‘cell-cell junction’ GO category (GO:0005911). During penetration of the uterine basal lamina by the trophoblasts, junctional complexes are formed between the syncytial trophoblast and maternal endothelial cells of blood vessels [1]. CHEM, by modulating the TGFβ1, EpCAM, ESAM, and PECAM1 proteins content in the endometrium, may control endothelial cell migration and tube formation intensity, which are crucial for endometrial cell differentiation during implantation. The above reports, together with the DRPs discussed in the previous paragraph, confirm the important role of CHEM in the endometrial remodeling and preparation for embryo implantation.

TGFβ1 and Vascular Cell Adhesion Molecule 1 (VCAM1) up-regulated in this study by CHEM, are both classified in the ‘leukocyte cell-cell adhesion’ category (GO:0007159). Both proteins play a key role in the activation of NK cells, which may have a direct impact on the pathogenesis of preeclampsia [59,73]. Preeclampsia is a common disorder that develops during pregnancy (4–5% of human pregnancies). It is characterized by elevated blood pressure and an intensified systemic inflammatory reaction. It has been suggested that the abnormal invasion of trophoblasts into the uterine blood vessels and the immunological intolerance between the fetal-placental and maternal tissues are responsible for the occurrence of this disease [74]. Recent reports confirmed the role of CHEM in the pathogenesis of preeclampsia. Tan et al. [75] indicated that placental CHEM expression and release increased in women with this disorder. It has been shown that CHEM reduced vascular endothelial factor-A and inhibited trophoblast migration and invasion. Moreover, according to the same authors, trophoblast CHEM overexpression in mice contributed to fetal growth restriction [75]. In the present study, CHEM, by increasing the expression of TGFβ1 protein in the endometrium, may contribute to the inadequate activation of NK cells and the development of preeclampsia. Cell adhesion molecules, due to their role in the adhesion of leukocytes to endothelial cells and the migration of leukocytes to the perivascular tissue, are believed to be subsequent indicators of endothelial dysfunction in preeclampsia [76]. VCAM1 has become the subject of many studies in terms of its involvement in the pathophysiology of preeclampsia. Various research suggests an increase in VCAM1 plasma concentration in preeclamptic patients compared with normal pregnancy [76,77,78,79]. It can be assumed that CHEM may regulate cytotrophoblast invasion in the spiral arteries and endothelial cell dysfunction during the pathogenesis of preeclampsia. Two other proteins associated with the development of preeclampsia and affected by CHEM in this study are Tubulointerstitial nephritis antigen-like 1 (TINAGL1) and Endoplasmic Reticulum Aminopeptidase 2 (ERAP2). TINAGL1 is a matricellular protein up-regulated in this study by CHEM interacting with structural matrix proteins and promoting cell adhesion [80]. The presence of the TINAGL mRNA and protein has been demonstrated in the mouse uterus [81] and embryos [82]. The expression of TINAGL1 was increased just before implantation in embryos and the postimplantation period in the decidual endometrium [81,82]. Mary et al. [83] reported that TINAGL1 was present in the human placenta and down-regulated under preeclamptic conditions. ERAP2 is known for its involvement in processes regulating immune recognition, angiogenesis, and blood pressure. Moreover, studies reported that ERAP2 abundance is lower in the placenta of women with preeclampsia [84,85]. Additionally, the reports of other teams also confirmed the correlation between the CHEM serum concentration with preeclampsia [86,87]. The above suggest CHEM involvement in the broadly understood pathogenesis of this disorder.

Considering the above-described roles of proteins differentially regulated under the influence of CHEM, we suggest that the adipokine takes part in the endometrial cell proliferation, migration and invasion at the time of implantation. It also regulates the endometrial immune response, sensitivity to P_4_, and the formation of new blood vessels_._ Additionally, CHEM appears to be an important factor involved in endothelial cell dysfunction during the pathogenesis of preeclampsia. Our results support the hypothesis that a balanced expression of CHEM is important for matrix remodeling and controlled trophoblast attachment during the implantation period. The obtained results provide a platform for a better understanding of the influence of CHEM on the porcine endometrium during implantation. However, future studies must be carried out to identify the exact mechanism of CHEM action.

## Data Availability

The mass spectrometry proteomics data have been deposited to the ProteomeXchange Consortium via the PRIDE [88] partner repository with the dataset identifier PXD031795. Other data are available on request from the corresponding author.

## Supplementary Materials

The following supporting information can be downloaded at: https://www.mdpi.com/article/10.3390/cells11071161/s1, Table S1. Total proteome changes in the porcine endometrial tissues induced by chemerin treatment; Table S2. The differentially regulated proteins in endometrial tissues after chemerin treatment; Table S3. Gene Ontology and Kyoto Encyclopedia of Genes and Genomes signaling pathways enrichment analysis results.
